# SARS-CoV-2 Nucleocapsid Protein Interacts with RIG-I and Represses RIG-Mediated IFN-β Production

**DOI:** 10.3390/v13010047

**Published:** 2020-12-30

**Authors:** Keli Chen, Feng Xiao, Dingwen Hu, Weiwei Ge, Mingfu Tian, Wenbiao Wang, Pan Pan, Kailang Wu, Jianguo Wu

**Affiliations:** 1 State Key Laboratory of Virology, College of Life Sciences, Wuhan University, Wuhan 430072, China; kelich@whu.edu.cn (K.C.); 2014202040014@whu.edu.cn (F.X.); 2018202040031@whu.edu.cn (D.H.); 2019202040020@whu.edu.cn (W.G.); 2015202040056@whu.edu.cn (M.T.); wukailang@whu.edu.cn (K.W.); 2 Guangdong Provincial Key Laboratory of Virology, Institute of Medical Microbiology, Jinan University, Guangzhou 510632, China; shabiao1212@whu.edu.cn (W.W.); doublepan@whu.edu.cn (P.P.); 3 Foshan Institute of Medical Microbiology, Foshan 528315, China

**Keywords:** coronavirus disease 2019, COVID-19, severe acute respiratory syndrome coronavirus 2, SARS-CoV-2, nucleocapsid, interferon, IFN, retinoic acid-inducible gene I, RIG-I

## Abstract

SARS-CoV-2 is highly pathogenic in humans and poses a great threat to public health worldwide. Clinical data shows a disturbed type I interferon (IFN) response during the virus infection. In this study, we discovered that the nucleocapsid (N) protein of SARS-CoV-2 plays an important role in the inhibition of interferon beta (IFN-β) production. N protein repressed IFN-β production induced by poly(I:C) or upon Sendai virus (SeV) infection. We noted that N protein also suppressed IFN-β production, induced by several signaling molecules downstream of the retinoic acid-inducible gene I (RIG-I) pathway, which is the crucial pattern recognition receptor (PRR) responsible for identifying RNA viruses. Moreover, our data demonstrated that N protein interacted with the RIG-I protein through the DExD/H domain, which has ATPase activity and plays an important role in the binding of immunostimulatory RNAs. These results suggested that SARS-CoV-2 N protein suppresses the IFN-β response through targeting the initial step, potentially the cellular PRR–RNA-recognition step in the innate immune pathway. Therefore, we propose that the SARS-CoV-2 N protein represses IFN-β production by interfering with RIG-I.

## 1. Introduction

Coronavirus disease 2019 (COVID-19), caused by a novel coronavirus known as severe acute respiratory syndrome coronavirus 2 (SARS-CoV-2), was identified as a pandemic by the World Health Organization (WHO) on 11 March 2020 [1]. The viral infection poses a huge threat to the global public health. Globally, as of 5:13 p.m. CET, 19 November 2020, there have been 55,928,327 confirmed cases of COVID-19, including 1,344,003 deaths, reported to the WHO. (https://covid19.who.int/). SARS-CoV-2 is an enveloped, non-segmented, positive-sense RNA virus in the *Coronaviridae* family, *Orthocoronavirinae* subfamily, *Betacoronaviruses* genus, and *Sarbecovirus* subgenus [2,3]. SARS-CoV-2 is a novel *β-coronavirus* and zoonotic coronavirus, following the previously identified respiratory syndrome coronavirus (SARS-CoV) and Middle East respiratory syndrome coronavirus (MERS-CoV) [4].

The viral genome sequence is about 29,903 nt and has 5′ and 3′ terminal sequences that are typical of *Betacoronaviruses* with a short untranslated (UTR) 5′ and 3′ terminus. The order of the genes (5′ to 3′) is replicase ORF1ab, spike (S), envelope (E), membrane (M), nucleocapsid (N), and accessory proteins ORF3a, ORF6, ORF7a, ORF7b, ORF8, ORF9a, and ORF9b [5,6]. ORF1ab is further cleaved into 15 nonstructural proteins (NSP1–10, 12–16) by its papain-like proteinase (NSP3) and 3C-like proteinase (NSP5) region [7]. Referring to SARS-CoV, the nucleocapsid protein is one of the most vital structural components that is bound to the nucleic acid material of the virus structurally. N protein binds to nucleic acid, and has an interaction with the M protein, which is involved in processes related to viral assembly, viral packaging during viral replication cycle, and the cellular response of host cells to viral infections [8,9,10]. All the functions and characteristics of SARS-CoV N protein also apply to all CoV N proteins. SARS-CoV N protein is also heavily phosphorylated and suggested to lead to enhancing the affinity for viral RNA by structural changes [11].

As the first line of defense against viruses, type I interferons (IFNs) play key roles in initiating host antiviral responses. As an early response to virus infection, the host immune system is triggered by viral components through the pathogen-associated molecular patterns (PAMPs), such as single-stranded RNA (ssRNA), double-stranded RNA (dsRNA), DNA, or glycoproteins, which are recognized by the pattern recognition receptors (PRRs) [12]. There are two major pathways for the activation of type I IFNs following RNA virus infection: the RIG-I-like receptors (RLRs) and the Toll-like receptors (TLRs) [13]. Previous studies have shown that RIG-I recognizes the 5′ ends of RNA molecules for several biochemical features: 5′-PPP RNA or RNAs with uncapped diphosphate (PP) groups, and the 5′-terminal nucleotide needs to be unmethylated at its 2′-O position [14]. RIG-I directly binds to viral 5′-PPP RNA or RNAs with uncapped diphosphate (PP) groups and short dsRNA through its helicase and repressor domain (RD), which are found in cells infected with a variety of RNA viruses [13,15]. RIG-I dephosphorylation occurs after recognition of viral RNA, which then triggers RIG-I polyubiquitination by ubiquitin E3 ligases tripartite-motif protein 25 (TRIM25) [16]. Polyubiquitinated RIG-I interacts with the mitochondrial antiviral-signaling protein (MAVS) and then initiate the antiviral signaling cascade through the N-terminal caspase recruitment domains (CARD) to CARD, activating TANK-binding kinase 1 (TBK1) and then activating the inhibitor of κB kinase-ε (IKKε), leading to the phosphorylation and activation of interferon regulatory factor 3 (IRF3) [17,18,19], and eventually leading to the production of type I interferons (IFNs). The interferon binds to their receptors and induce interferon-stimulated genes (ISGs), which result in antiviral responses [20]. In turn, coronaviruses have evolved strategies to overcome interferons. Humans have been infected by coronaviruses since the 1960s, which causes only common respiratory diseases [4]. Like most other viruses, the main purpose of coronaviruses is to establish infection in the host, propagate and spread within the host, and transmit virus progenies to new hosts. Many studies have demonstrated that coronaviruses antagonize interferons in different ways. SARS-CoV accessory proteins ORF8b/ab suppresses the interferon signaling pathway by mediating ubiquitin-dependent rapid degradation of IRF3 [21]. SARS-CoV ORF3b, ORF6, and N proteins antagonize interferons by different mechanisms [22]. Porcine epidemic diarrhea virus (PEDV) N protein antagonizes IFN-β production by sequestering the interaction between IRF3 and TBK1 [19]. The SARS-CoV N protein inhibits type I interferon production by interfering with TRIM25-mediated RIG-I ubiquitination [23]. SARS-CoV N protein targets the initial step, probably the cellular PRR–RNA-recognition step in the innate immune pathways to suppress the IFN expression responses, with the domain of the N protein being critical for its antagonism of IFN induction [24]. Both mouse hepatitis virus (MHV) and SARS-CoV N proteins can perturb the function of cellular protein activator of protein kinase R (PACT) to circumvent the innate antiviral response [25]. Porcine deltacoronavirus (PDCoV) N protein suppressed IFN-β production by interfering with porcine the RIG-I dsRNA-binding and K63-linked polyubiquitination [26]. PDCoV N protein antagonizes IFN-β production by impairing dsRNA and PACT binding to RIG-I [27]. MERS-CoV N protein suppresses type I and type III interferon induction by targeting RIG-I signaling [28]. Given SARS-CoV-2 is highly infectious, it must possess mechanisms suppressing innate immune responses. Recent studies have shown that coronavirus can cause substantial but delayed IFN-β levels, and ORF6 inhibits type I IFN production and downstream signaling [6]. SARS-CoV-2 N protein antagonizes type I interferon signaling by suppressing phosphorylation and nuclear translocation of STAT1 and STAT2 [29]. It is reported that several SARS-CoV-2 proteins, such as ORF3b, ORF6, ORF7b, M, N, nsp1, nsp6, and nsp13, could antagonize the interferon response to varying degrees in different ways [30]. In this study, our finding demonstrates a new mechanism evolved by SARS-CoV-2 to circumvent the host’s antiviral immunity. We found that the structural protein N of SARS-CoV-2 antagonizes IFN-β induced upon SeV infection and by poly (I:C) treatment, and represses the IFN-β production induced by the RIG-I pathway signaling molecules, namely, RIG-I, MAVS, TBK1, and IKKε. Moreover, N protein affects the process of IRF3 phosphorylation and transferring to the nuclear. We noted that N protein interacted with RIG-I through the DExD/H domain. Therefore, these data imply that the inhibition of IFN-β production by the N protein may be due to the ability of the N protein binding to and shielding viral RNAs from being recognized by cellular PRRs. Our study is the first investigation that shows the interaction between SARS-CoV-2 and RIG-I, which provides insights into the mechanism of SARS-CoV-2 antagonizing innate immunity.

## 2. Materials and Methods

### 2.1. Cell Lines and Cultures

A549 (ATCC, #CCL-185), HeLa (ATCC, #CCL-2), and HEK293T cells were purchased from the American Type Culture Collection (ATCC, Manassas, VA, USA). A549, Hela, and HEK293T cells were cultured in Dulbecco’s modified Eagle’s medium (DMEM) (Gibco, Grand Island, NY, USA) supplemented with 10% FBS, 100 U/mL penicillin, and 100 μg/mL streptomycin sulfate. All these mentioned cells were maintained at 37 °C in 5% CO_2_ incubator.

### 2.2. Antibodies and Reagents 

Rabbit anti-HA (H6908) (1:1000) and Mouse anti-Flag (F1804) (1:2000) antibodies were purchased from Sigma-Aldrich (St Louis, MO, USA). Monoclonal mouse anti-GAPDH (1E6D9) (1:5000) were purchased from Proteintech Group (Wuhan, China). Rabbit anti-LaminA/C (10298-1-AP) (1:3000) were purchased from Proteintech Group (Wuhan, China). Antibody anti-p-IRF3 at Ser396 (4947s) (1:1000) was purchased from Cell Signaling Technology (CST, Boston, MA, USA). Antibody anit-IRF3 (11312-1-AP) (1:1000) was purchased from Proteintech Group (Wuhan, China). Antibody anti-RIG-I (D1466) (1:1000) was purchased from Cell Signaling Technology (Beverly, MA, USA). The poly(I:C) was purchased from InvivoGen (San Diego, CA, USA). Lipofectamine 2000 were purchased from Invitrogen Corporation (Carlsbad, CA, USA). DMEM were purchased from Gibco (Grand Island, NY, USA). Protein ladder (26616) was purchased from Thermo Scientific (Rockford, IL, USA). Complete, EDTA-free Protease Inhibitor Cocktail was purchased from Roche (Basel, Switzerland).

### 2.3. Plasmids Construction and Primers

IFN-β-Luc reporter plasmids were gifted from Dr. Ying Zhu of Wuhan University, China. Mammalian expression plasmids for HA- and Flag-N were constructed by the standard molecular cloning method from the temple pcDNA3.1(+)-HA-N, which is synthesized from GeneScript (Nanjing, China) and the GenBank accession number is MN908947.3. Mammalian expression plasmids for HA-, Flag- RIG-I, or Flag-tagged MAVS, TBK1, IKKε, and IRF3 were constructed by the standard molecular cloning method from cDNA templates. The expression plasmids CARD domain (1–256 aa), DExD/H-box domain (257–735 aa), and repressor domain (RD) (736–925 aa) of the RIG-I protein were all cloned into pcAGGS-HA vector. 

### 2.4. Quantitative RT-PCR Analysis and Primers

Total RNA was extracted from target cells using TRIzol reagent (Invitrogen Life Technologies; Carlsbad, CA, USA) according to the manufacturer’s instructions. A total of 1 μg of RNA was used to synthesize the complementary DNA by HiScript II qRT Supermix (Vazyme Biotech Co, Nanjing, China), and the following step regarding the quantitative real-time analysis was performed by using ChamQ SYBR qPCR Master Mix (Vazyme Biotech Co, Nanjing, China) on a Roche LC480 (Roche Diagnostics, Penzberg, Germany). All specific primers for testing were designed by Primer Bank or Primer Premier 5.0. The reaction mixture consisted 10 μL of SYBR Green PCR master mix, 1 μL of DNA template obtained in the previous step, 1 μL of primers (10 μM), and 8 μL of RNase-free water. The data represent relative numbers of mRNA copies normalized to GAPDH, used as a reference gene, and the gene expression levels were calculated using the 2^−ΔΔ*CT*^ method. The primer sequences were as follows: IFN-β forward: 5′-TGGGAGGCTTGAATACTGCCTCAA-3′; and IFN-β reverse: 5′-TCCTTGGCCTTCAGGTAATGCAGA-3′, the amplicon size: 336 [31]. GAPDH forward: 5′-ATGACATCAAGAAGGTGGTG-3′; GAPDH reverse: 5′-CATACCAGGAAATGAGCTTG-3′, the amplicon size: 179 [32].

### 2.5. Luciferase Reporter Assays

In a 24-well plate, HEK293T cells were transfected with 200 ng luciferase reporter (*Firefly* luciferase) and 20 ng pRL-TK (*Renilla* luciferase plasmid) when 60–70% confluent, together with an indicated variety expression plasmid or empty vector plasmid. HEK293T cells were co-transfected with HA-N or empty vector plasmid for 24 h and then infected with SeV for 16 h or stimulated with poly(I:C) for 16 h. Luciferase activity was represented by the relative firefly luciferase activity normalized to the *Renilla* luciferase activity, which was measured by using a Dual-Luciferase Reporter Assay System kit (Promega, San Luis Obispo, CA, USA) according to the manufacturer’s protocol.

### 2.6. Western Blotting and Co-Immunoprecipitation Assay

Cells were lysed by a lysis buffer (50 mM Tris-HCl, 300 mM NaCl, 1% Triton-X, 5 mM EDTA, and 10% glycerol, pH7.5) in the presence of the cocktail (Roche) on ice. The protein concentration was determined by Bradford assay (Bio-Rad, Hercules, CA, USA). After adding the 5× SDS loading buffer, cell lysates (30 μg) were electrophoresed in a 10–12% SDS-PAGE gel and then transferred to a PVDF membrane (Millipore, MA, USA) for 2 h. The PVDF membranes were blocked with 5% skim milk in phosphate-buffered saline (PBS) with 0.05% Tween 20 (PBST) before being incubated with the antibody. The membranes were then incubated with specific primary antibodies overnight at 4 °C. The next day, after being incubated with the secondary antibodies at room temperature for 1 h and following a wash with the PBST, the protein bands were detected by using the Clarity Western ECL substrate (Bio-Rad) and Luminescent Image Analyzer (LAS-4000, Fujifilm, Tokyo, Japan). 

For Co-IP assays, cells were washed with PBS and then lysed in a lysis buffer (50 mM Tris-HCl, pH7.5, 300 mM NaCl, 1% Triton-X, 5 mM EDTA, and 10% glycerol) added with the protease inhibitors. Of the lysates, 80 μL were reserved for immunoblot analysis to detect the expression of the target proteins. The rest of the lysates were incubated with a control IgG or the indicated primary antibodies at 4 °C overnight and were further incubated with protein A/G-agarose (GE Healthcare, Waukesha, WI, USA) for 2 h the next day. The beads were washed four times in a washing buffer (50 mM Tris-HCl, pH7.4, 150 mM NaCl, 1% Triton-X, 5 mM EDTA, and 10% glycerol) and then were resuspended in 60 μL 2× SDS loading buffer for Western blot analysis. 

### 2.7. Nuclear and Cytoplasmic Extraction

In a 6-well plate, 60–70% confluent HEK293T cells were transfected with the indicated plasmids for 24 h, and then disposed by using the nuclear and cytoplasmic extraction reagents according to the manufacturer’s instructions. (Thermo Scientific, 78833, USA). The cytosol or nuclear lysate concentration was determined by Bradford assay (Bio-Rad, Hercules, CA, USA).

### 2.8. Immunofluorescence Analysis

Around 40–50% HEK293T cells or Hela cells were transfected with the indicated plasmids (500 ng) for 24 h, and cells were then washed with PBS and then fixed in 4% paraformaldehyde at room temperature for 15 min, followed by permeabilizing with the wash buffer (PBS containing 0.1% Triton X-100) for 5 min, washed three times with PBS containing 0.1% BSA, and finally blocked with PBS containing 5% BSA for 1 h. The cells were incubated with the primary antibody overnight at 4 °C subsequently, followed by incubation with FITC-conjugate goat anti-rabbit IgG and Dylight Cy3-conjugate goat anti-mouse IgG (Proteintech) for 1 h. Using the wash buffer three times, cells were incubated with DAPI solution for 5 min, and then washed three more times with wash buffer and PBS. Finally, the cells were analyzed by using a confocal laser scanning microscope (Fluo View FV1000; Olympus, Tokyo, Japan).

### 2.9. Statistical Analyses

All experiments were repeated at least three times with similar results. Samples were analyzed by the Student’s *t*-test for two groups and one-way ANOVA for multiple groups (GraphPad Prism 6.0, Inc., La Jolla, CA, USA). A Levene’s test was performed to determine the equality of the variances, which provided the information needed for the Student’s *t*-tests regarding the equality of the means. Means were illustrated using histograms, with error bars representing the standard error of the mean (SEM); values of *p* < 0.05 were considered to indicate statistical significance (ns, there was no significant difference, * *p* < 0.05, ** *p* < 0.01, and *** *p* < 0.001). 

## 3. Results

### 3.1. SARS-CoV-2 N Protein Represses IFN-β Production Induced by Poly(I:C) and Sendai Virus

**Figure 1:**
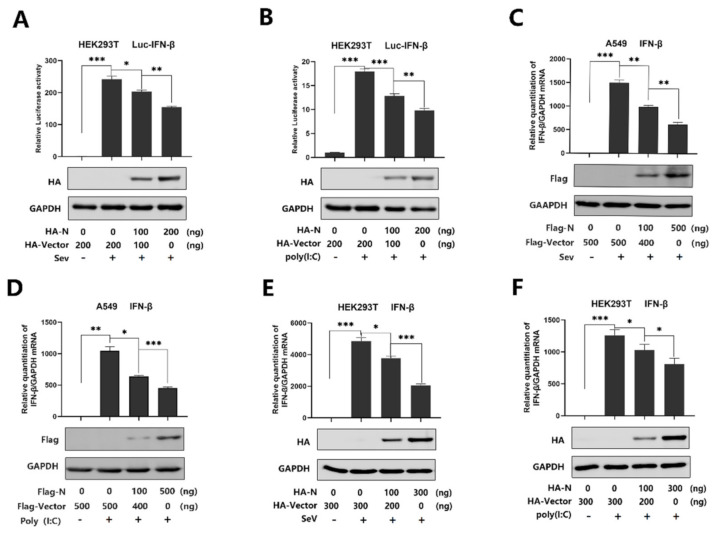
SARS-CoV-2 N protein represses IFN-β production induced by poly(I:C) and Sendai virus. (**A**,**B**) HEK293T cells were transfected with IFN-β luciferase reporter pIFN-β-Luc and pPRL-TK for 24 h and then infected with SeV (0.1 MOI) for 16 h (**A**) or transfected with cytoplasmic poly(I:C) (2 μg/mL) for 16 h (**B**). Cell lysates were harvested, IFN-β-Luc reporter activity was determined by dual luciferase reporter assays (top), and HA-N was detected by Western blotting (bottom). (**C**,**D**) A549 cells were transfected with pFlag-N for 24 h and infected with SeV (MOI = 0.1) for 16 h (**C**) or transfected with poly(I:C) (1 μg/mL) for 16 h (**D**). IFN-β mRNA was determined by q-PCR (top) and Flag-N was confirmed by Western blotting (bottom). (**E**,**F**) HEK293T cells were transfected with pHA-N for 24 h and infected with SeV (MOI = 0.1) for 16 h (**E**) or transfected with poly(I:C) (2 μg/mL) for 16 h (**F**). IFN-β mRNA was determined by q-PCR (top) and HA-N was confirmed by Western blotting (bottom). Data in A–F were expressed as the mean ± SEM of at least three independent experiments. * *p* < 0.05, ** *p* < 0.01, *** *p* < 0.001.

SARS-CoV-2 infection causes clinical symptoms of varying degrees, with both mild and severe infection accompanied by disturbance of the type I interferon [33]. IFN-β plays an important role in activating an immune response and suppressing virus replication [34,35,36,37]. However, studies have shown that SARS-CoV N protein disturbed the IFN-β levels [22,23,24], and recent studies have suggested that SARS-CoV-2 caused a delayed interferon response [6]. Indeed, SARS-CoV-2 N protein plays a key role in viral genome packaging and the replication cycle [38,39]. Here, we explored the relationship between SARS-CoV-2 N protein and IFN-β response. We initially showed that the IFN-β-Luc activity was induced upon SeV infection (Figure 1A) or by poly(I:C) treatment (Figure 1B), but the induction was attenuated by N protein in the HEK293Tcells (Figure 1A,B). Additionally, IFN-β mRNA was significantly induced by SeV infection (Figure 1C) and poly(I:C) treatment (Figure 1D), but such an induction was suppressed by the N protein in A549 cells (Figure 1C,D). Moreover, the same inhibition was observed in the HEK293T cells (Figure 1E,F). These results demonstrate that SARS-CoV-2 N protein suppresses IFN-β expression upon the infection of SeV or by the stimulation of poly(I:C).

### 3.2. SARS-CoV-2 N Protein Represses IFN-β Production by Targeting the RIG-I Pathway

**Figure 2:**
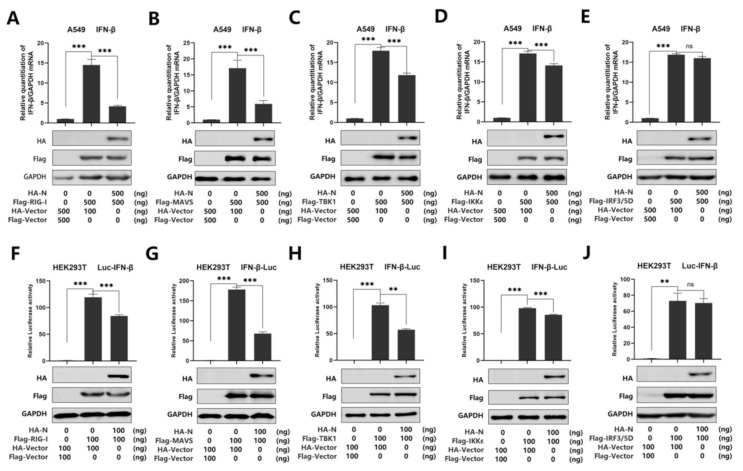
SARS-CoV-2 N protein represses IFN-β production by targeting the RIG-I pathway. (**A**–**E**) A549 cells were co-transfected with pHA-N, together with pFlag-RIG-I (**A**) or pFlag-MAVS (**B**), pFlag-TBK1 (**C**), pFlag-IKKε (**D**), of pFlag-IRF3/5D (**E**) for 24 h. Cell lysates were harvested, IFN-β mRNA was determined by q-PCR (top), and HA-N, Flag-RIG-I (**A**), MAVS (**B**), TBK1 (**C**), IKKε (**D**), and pFlag-IRF3/5D (**E**) were confirmed by Western blotting (bottom). (**F**–**J**) HEK293T cells were co-transfected with pIFN-β-Luc, pPRL-TK, and pHA-N, together with pFlag-RIG-I (**F**), pFlag-MAVS (**G**), pFlag-TBK1 (**H**), pFlag-IKKε (**I**), and pFlag-IRF3/5D (**J**) for 24 h. Cell lysates were harvested, IFN-β-Luc reporter activity was determined by dual luciferase reporter assays (top), and HA-N, Flag-RIG-I (**F**), or MAVS (**G**), TBK1(**H**), IKKε(**I**), and pFlag-IRF3/5D (**J**) were confirmed by Western blotting (bottom). Data in A–F were expressed as the mean ± SEM of at least three independent experiments. ns, there was no significant difference, ** *p* < 0.01, *** *p* < 0.001.

Recent studies have shown that SARS-CoV-2 N protein plays a key role in viral genome packaging [38,39]. Indeed, RIG-I is a key sensor of RNA virus infection, which mediates the activation of type I interferons and other genes that collectively establish an antiviral host response. Moreover, it has been reported that RIG-I and melanoma differentiation-associated gene-5 (MDA5) are the main sensors for the identification of a coronavirus [14]. Studies have also shown that MERS-CoV N protein suppresses RIG-I-CARD-induced, but not MDA5-CARD-induced, IFN-β promoter activity [28]. Another study also proved that the MERS-CoV and SARS-CoV N protein interacted with TRIM25 and inhibited RIG-I signaling [23]. In addition, it is reported that swine acute diarrhea syndrome coronavirus (SADS-CoV) antagonizes interferon-β production via blocking MAVS and RIG-I [40]. Overall, previous studies indicate that RIG-I plays an important role in coronavirus recognition. To further verify the inhibitory effect of N protein on IFN-β activation, we investigated whether N protein affects RIG-I signaling. We used real-time PCR and luciferase reporter assays to analyze the IFN-β mRNA level. As expected, IFN-β mRNA was induced upon overexpression of RIG-I, MAVS, TBK1, IKKε, and IRF3/5D, while such an induction was attenuated by N protein but not its activation induced by IRF3/5D in A549 cells (Figure 2A–E). Meanwhile, we found that overexpression of N protein attenuated the activation of IFN-β promoter luciferase reporter activity by over-expressed RIG-I, MAVS, TBK1, and IKKε, while N protein did not interfere with the activity of IFN-β promoter stimulated by IRF3/5D in HEK293T cells (Figure 2F–J). Taken together, we demonstrated that SARS-CoV-2 N protein suppresses IFN-β production by repressing the RIG-I pathway.

### 3.3. SARS-CoV-2 N Protein Restricts IRF3 Phosphorylation and Nuclear Translocation

**Figure 3:**
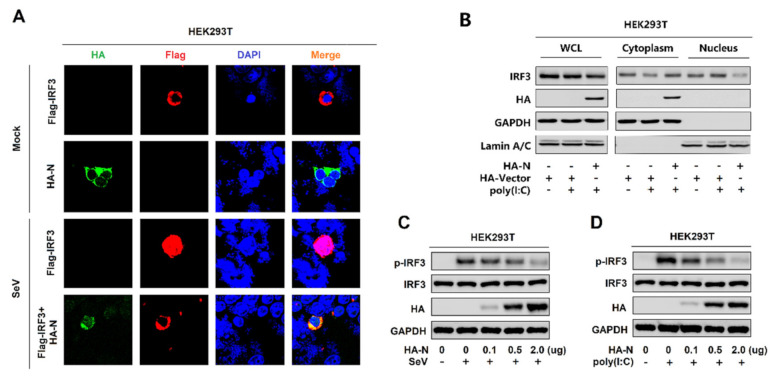
SARS-CoV-2 N protein restricts IRF3 phosphorylation and nuclear translocation. (**A**) HEK293T cells were transfected with pFlag-IRF3 or pHA-N or co-transfected with pFlag-IRF3 and pHA-N for 24 h and infected with SeV (MOI = 0.1) for 16 h. The sub-cellular localizations of Flag-IRF3 (red), HA-N (green), and nucleus marker DAPI (blue) were analyzed by confocal microscopy. (**B**) HEK293T cells were transfected with pHA-N for 24 h and treated with poly(I:C) (2 μg/mL) for 16 h. IRF3 in the nuclear fractions or the cytoplasmic was determined by immunoblotting analyses. GAPDH served as a cytoplasmic control and Lamin A/C served as a nuclear protein control. (**C**,**D**) HEK293T cells were transfected with pHA-N for 24 h and (**C**) infected with SeV (MOI = 0.1) for 16 h, (**D**) treated with poly(I:C) (2 μg/mL) for 16 h. Phosphorylated IRF3, total IRF3, and GAPDH were subjected to Western blotting with the indicated antibodies anti-pIRF3, anti-IRF3, anti-HA, and anti-GAPDH, respectively.

IRF3 is an important component of the RIG-I pathway. The activated IRF3 is characterized by phosphorylation, which leads to IRF3 nuclear localization and IFN-β production [41]. Here, we explored the effect of N protein on IRF3 phosphorylation and nuclear translocation. In mock-infected HEK293T cells, IRF3 alone was mainly distributed in the cytoplasm, and N was also located in the cytoplasm. However, in SeV-infected HEK293T cells, IRF3 was translocated from the cytoplasm to the nucleus in the absence of N protein; interestingly, most of IRF3 remained in the cytoplasm in the presence of N (Figure 3A). In addition, poly(I:C)-induced IRF3 nuclear translocation was disturbed by the overexpression of N protein in HEK293T cells. In the cytoplasmic extraction part of the HEK293T cells, poly(I:C)-induced IRF3 was enhanced by N protein; while, in the nuclear extraction part of the HEK293T cells, poly(I:C)-induced IRF3 was attenuated by N protein (Figure 3B). IRF3 phosphorylation was induced upon SeV infection (Figure 3C) or being transfected with cytoplasmic poly(I:C) (Figure 3D) in HEK293T cells, whereas N protein significantly repressed SeV or poly(I:C)-induced IRF3 phosphorylation but not IRF3 production (Figure 3C,D). Taken together, these results reveal that N protein restricts IRF3 phosphorylation and nuclear translocation, thus repressing IRF3 activation. However, the IRF3 production is not impacted.

### 3.4. SARS-CoV-2 N Protein Binds to the DExD/H Domain of RIG-I

**Figure 4:**
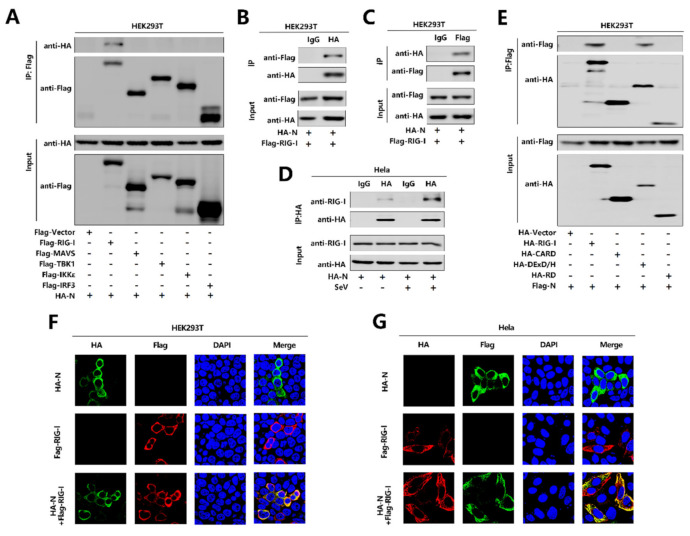
SARS-CoV-2 N protein binds to the DexD/H domain of RIG-I. (**A**) HEK293T cells were co-transfected with pHA-N in combination with pFlag-Vector, pFlag-RIG-I, pFlag-MAVS, pFlag-TBK1, pFlag-IKKε, or pFlag-IRF3. Cell lysates were subjected to immunoprecipitation (IP) using anti-Flag antibody and analyzed by immunoblotting using anti-HA and anti-Flag antibody (top). Cell lysates (30 μg protein) were analyzed directly by immunoblotting using anti-HA and anti-Flag antibody as input (bottom). (**B**,**C**) HEK293T cells were co-transfected with pHA-N and pFlag-RIG-I. Cell lysates were subjected to IP using control IgG or anti-HA antibody (**B**) and control IgG or anti-Flag antibody (**C**). Cell lysates (30 μg protein) were analyzed directly by immunoblotting using anti-HA and anti-Flag antibody as input (bottom). (**D**) Hela cells were transfected with pHA-N or transfected with pHA-N and infected with SeV (MOI = 0.1) for 16 h. Cell lysates were subjected to IP using control IgG and anti-HA antibody and then analyzed by immunoblotting using anti-HA antibody and anti-RIG-I antibody. Cell lysates (30 μg protein) were analyzed by immunoblotting using anti-HA and anti-RIG-I antibody as input (bottom). (**E**) HEK293T cells were co-transfected with pFlag-N in combination with pHA-Vector, pHA-RIG-I-(2CARD), pHA-DExD/H, or pHA-RD. Cell lysates were subjected to IP using anti-Flag antibody and then analyzed by immunoblotting using anti-HA and anti-Flag antibody (top). Cell lysates (30 μg protein) were analyzed directly by immunoblotting using anti-HA and anti-Flag antibody as input (bottom). (**F**,**G**) HEK293T cells (**F**) and Hela cells (**G**) were transfected with pHA-N or pFlag-RIG-I, or co-transfected with pFlag-RIG-I and pHA-N. The sub-cellular localizations of Flag-RIG-I (red), HA-N (green), and nucleus marker DAPI (blue) were analyzed by confocal microscopy.

To further explore the relationship between N protein with the RIG-I pathway, initially, we performed a co-immunoprecipitation (Co-IP) assay to determine whether N protein interacts with the RIG-I signaling components. Interestingly, N interacted with RIG-I but failed to interact with MAVS or TBK1, IKKε, and IRF3 (Figure 4A). We further confirmed that N protein associated with RIG-I and RIG-I interacted with N protein in the Co-IP assay in HEK293T cells (Figure 4B,C). Moreover, N protein was co-immunoprecipitated with endogenous RIG-I in Hela cells transfected with pHA-N, and the interaction was significantly enhanced upon SeV infection (Figure 4D). Furthermore, immunofluorescence analysis showed that RIG-I and N co-located in the cytoplasm in HEK293T cells (Figure 4F) and Hela cells (Figure 4G). Since RIG-I contains three domains, CARD, DExD/H, and RD [17], we explored which domain is involved in the interaction with N protein. To find this information, we performed a Co-IP assay and revealed that N protein interacted with the RIG-I and DExD/H domain, but not with the CARD or RD domain. Therefore, the results demonstrate that N binds to the DExD/H domain of RIG-I. 

## 4. Discussion

The recent emergence of the SARS-CoV-2 represents a global crisis, posing a great risk to human life and health. SARS-CoV-2 presents with a spectrum of clinical phenotypes, with most patients presenting mild-to-moderate symptoms, and 15% progressing typically to severe or critical disease within a week, and a small number of those develop acute respiratory disease syndrome (ARDS) requiring mechanical ventilation [42]. The elderly and individuals with underlying medical comorbidities, such as cardiovascular disease, chronic lung disease, diabetes mellitus, chronic kidney disease, hypertension, obesity, or cancer, have a much higher mortality rate than healthy young adults [43]. The underlying causes are unknown, but maybe due to an impaired interferon response and abnormal inflammatory responses observed in other zoonotic coronavirus infections, such as SARS -CoV and MERS-CoV [44].

SARS-CoV-2 appears to spread more efficiently compared to MERS-CoV or SARS-CoV, making it difficult to control and increasing its pandemic potential [45,46]. SARS-CoV-2 shows asymptomatic or mild symptoms while being contagious could be key factors for achieving such efficient transmission. To devise treatment strategies against SARS-CoV-2 infection, it is crucial to develop a comprehensive understanding of how the coronavirus hijacks the host in the process of infection [47].

As an important part of innate immunity, type I interferon plays a very important role in early response to virus infection, especially respiratory viruses [4]. Disorder in the type I interferon response may lead to serious pathological features. Previous studies have shown that neither SARS-COV nor MERS-CoV caused strong IFN expression in the innate immune response [23]. Using a SARS-CoV animal model, it was reported that a rapid and robust virus replication with delayed IFN-I can lead to pulmonary immunopathology, with fatal consequences [48]. Recent studies have shown consistent results that SARS-CoV-2 caused a delayed interferon response [6,49,50,51]. This may be the reason of the severe lung damage caused by the SARS-CoV-2. However, recent studies confirmed the potential for IFNs to enhance expression of host angiotensin-converting enzyme 2 (ACE2), suggesting that IFN treatment or natural co-infection may aggravate COVID-19 by up-regulating this critical virus entry receptor [52,53]. However, IFN is protective if used early after infection. At a later point in time, when the IFN and inflammatory cytokines are pathogenic, reducing the IFN and/or inflammatory cytokines, inhibiting the IFN and cytokine signaling, or reducing the IFN and cytokine production cells may be an effective therapeutic option to restore the balance of the exaggerated immune response [4]. Nevertheless, there is still a lot of in-depth research needed to find the specific interaction mechanism between SARS-CoV-2 and IFN.

In this study, we found that SARS-CoV-2 N protein significantly inhibited the mRNA levels of IFN-β induced by both poly(I:C) and SeV stimulation. In addition, N protein affected the phosphorylation and nuclear translocation of transcription factor IRF3, which is essential for IFN-β production upon virus infection, further indicating that N protein plays an important role in antagonizing the IFN-β response. It was reported that SARS-CoV N protein and MERS-CoV N protein impede RIG-I ubiquitination and activation and inhibit the phosphorylation of IRF3 [23,28]. PEDV N protein targets TBK1 by direct interaction, and this binding sequesters the association between TBK1 and IRF3, which in turn inhibits both IRF3 activation and type I IFN production [19]. SARS-CoV and MERS-CoV belong to the *β-coronavirus* genus, while PEDV belongs to the *α-coronavirus* genus, nevertheless, the N proteins show similar inhibitory effects on IRF3 phosphorylation and interferon production. In our study, similar results were obtained that SARS-CoV-2 N protein inhibits IRF3 activation. However, the molecular mechanisms involved are distinct. RIG-I is a key receptor for the recognition of RNA viruses and, interestingly, we found that N protein inhibits the IFN-β responses induced by overexpression of the RIG-I pathway signaling molecule, such as MAVS, TBK1, and IKKε, but not IRF3/5D. These results suggest that N antagonize IFN-β production by targeting IRF3 (before IRF3 activation) or another component upstream. The co-immunoprecipitation assay show that N protein interacts with RIG-I and failed to interact with several other components of the RIG-I pathway. These results suggest that IFN-β induced by overexpression of MAVS, TBK1, and IKKε may be affected at the initial step by the interaction of N protein with RIG-I; this is because IFN-β production requires recognition of the PAMP in the downstream signaling cascade. Moreover, it can be conceived that MAVS could be targeted as this latter protein interacts with RIG-I, which interacts with N. However, TBK1 activity can be regulated in a variety of ways, such as phosphorylation, ubiquitination, kinase activity modulation, and prevention of functional TBK1-containing complex formation. Similarly, IKKε activation also requires post-translational modification, such as ubiquitination and functional IKKε-containing complex assembly [54,55,56,57]. It is possible that N directly interferes with their activation step. Overall, the specific mechanism remains to be explored. It is reported that the PEDV N protein targets TBK1 by direct interaction and inhibits both IRF3 activation and type I IFN production [19]. This result is consistent with our study that SARS-CoV-2 N protein significantly impedes the activation of the IFN-β production, stimulated by TBK1. However, there is no interaction between SARS-CoV-2 N protein and TBK1. This difference may be due to the distant genetic relationship between the two viruses. Overall, it remains to be explored whether N protein directly affects the downstream molecules of the RIG-I pathway. Previous studies have shown that RIG-I plays a key role in identifying SARS-CoV and inducing IFN-β responses [14,23]. This is consistent with our research. We further revealed that SARS-CoV-2 N protein and RIG-I co-localized, and N protein interacted with RIG-I through the DExD/H domain of RIG-I. The DExD/H domain is the enzymatic active domain in RIG-I, which has ATPase activity, and ATP hydrolysis determines the dissociation rate of RIG-I from RNA, playing an important role in binding immunostimulatory RNAs [58,59,60,61]. This result is consistent with the function of RIG-I. It is reported that the coronaviruses N protein phosphorylation allows recruitment of the RNA helicase DDX1 to the phosphorylated-N-containing complex, which facilitates template readthrough and enables longer several subgenomic mRNA (sgmRNA) synthesis [62]. While we did find that SARS-CoV-2 N protein interacts with the DExD/H domain of RIG-I and represses RIG-mediated IFN-β production, interestingly, both the DExD/H domain of RIG-I and DDX1 helicase belong to the DEAD box helicase family. It implies that the coronavirus N protein can interact with the host through different mechanisms; i.e., establish infection in the host, propagate, and spread within the host.

According to previous studies on coronavirus, there are many strategies for virus escape innate immunity, such as residing in the coronavirus replication complex, using early endonuclease, or forming double-membrane vesicles to block RLRs recognition, thereby inhibiting the production of interferons and, moreover, suppressing RNase-L and host translation in infected cells [63]. On the other hand, the antigenic variation may be an effective mechanism for SARS-CoV to escape innate immune response. In fact, previous studies have suggested that the aa substitution in SARS-CoV M protein has been implicated in decreasing the level of IFN-β, and such an alteration is sufficient to abolish M-mediated IFN-β induction [64,65]. A study demonstrated that SARS-CoV N protein dramatically inhibited expression from an NF-κB responsive promoter and interferon-stimulated response element (ISRE) promoter after infection with SeV, as well as IRF3—the key protein necessary for the expression of IFN-β—therefore inhibiting the expression of IFN-β [22]. In another study, the SARS-CoV N protein was found to bind to tripartite motif protein 25 (TRIM25), inhibiting TRIM25-mediated RIG-I ubiquitination and activation and then affecting the interferon response [23]. A recent study found that SARS-CoV-2 N protein could have a physical association with stress granule protein G3BP1 (the Ras-GTPase-activating protein SH3-domain-binding protein 1) through affinity mass spectrometry (AP-MS); that is, a reliable interaction relationship [47]. Meanwhile, studies have found that G3BP1 serves as a positive regulator of the RIG-I-mediated signaling pathway. G3BP1 can co-locate with RIG-I and significantly improve the synthesis of IFN-β induced by RIG-I. This may be a potential mechanism by which the SARS-CoV-2 N protein affects IFN-β via the RIG-I signaling pathway [66]. In addition, different cell types display different responses to the coronavirus infection. It has been reported that plasmacytoid dendritic cells (pDCs) induce robust expression of IFN-I after coronavirus infection, which utilized TLR7 to sense the viral nucleic acids and then induced the robust expression of IFN-I [67]. Therefore, individual performance should be much more accounted for in further research.

In conclusion, our study provides insights into the mechanism of SARS-CoV-2 antagonism against interferons, which is conducive to the subsequent development of a relevant effective therapy, and also helps to provide ideas for research about other new viruses. However, the specific mechanism by which SARS-CoV-2 affects the interferon pathway through RIG-I remains to be explored. In addition, it is possible that other SARS-CoV-2 proteins can inhibit or promote interferon production and there may be other PRRs to monitor and interplay with SARS-CoV-2. All these aspects need to be explored in future work.

## Data Availability

All the data used in this study is already provided in the manu-script at its required sections. There is no underlying data available.
